# Cross-sectional and longitudinal associations between receptive arts engagement and loneliness among older adults

**DOI:** 10.1007/s00127-019-01764-0

**Published:** 2019-09-11

**Authors:** Urszula Tymoszuk, Rosie Perkins, Daisy Fancourt, Aaron Williamon

**Affiliations:** 1 Centre for Performance Science, Royal College of Music, London, UK; 2 Faculty of Medicine, Imperial College London, London, UK; 3 Department of Behavioural Science and Health, University College London, London, UK

**Keywords:** Ageing, Arts, Cohort study, Cultural engagement, ELSA, Perceived isolation

## Abstract

**Purpose:**

Loneliness in older adulthood is a societal and public health challenge warranting identification of sustainable and community-based protective factors. This study investigated whether frequency of receptive arts engagement is associated with lower odds of loneliness in older adults.

**Methods:**

We used data of respondents from waves 2 (2004–2005) and 7 (2014–2015) of the English Longitudinal Study of Ageing (ELSA) and examined cross-sectional (*n* = 6222) and longitudinal (*n* = 3127) associations between frequency of receptive arts engagement (including visits to the cinema, museums/galleries/exhibitions, theatre/concerts/opera) and odds of loneliness (cut-off ≥ 6 on three-item short form of the Revised UCLA Loneliness Scale). We fitted logistic regression models adjusted for a range of sociodemographic, economic, health and social, community and civic engagement factors.

**Results:**

Cross-sectionally, we found dose–response negative associations between engagement with all receptive arts activities and odds of loneliness. Prospectively, in the fully-adjusted models we found most robust evidence for the negative association between engagement with museums/galleries/exhibitions and odds of loneliness (OR = 0.68, 95% CI 0.48–0.95) for those who engaged every few months or more often compared with those who never engaged. We found weaker evidence for lower odds of loneliness for more frequent engagement with theatre/concerts/opera.

**Conclusions:**

Frequent engagement with certain receptive arts activities and venues, particularly museums, galleries and exhibitions, may be a protective factor against loneliness in older adults. Future research is needed to identify the mechanisms through which this process may occur, leading to better understanding of how arts activities and venues can reduce loneliness among older adults.

## Introduction

Recent research has shown that one in nine people aged 55 years and over living in England between 2016 and 2017 reported feeling lonely always or often, and an additional four in nine reported feeling lonely some of the time [1]. Concurrently, the total population of adults aged 55 years and above living in the UK is predicted to increase, from over 19 million in 2016 to over 26 million (i.e. more than one in three people) by 2040 [2]. As loneliness and inadequate social relationships have been previously linked with, for instance, increased risk of depressive symptoms [3], coronary heart disease and stroke [4], and mortality [5], this demographic change combined with vulnerability to loneliness among older adults poses a significant societal and public health challenge.

The risk of loneliness increases with age due to factors such as bereavement, decline in physical health, cognitive function and mobility as well as changes in living and socioeconomic circumstances, including living alone or in a nursing home [6–9]. Previous research also highlights the significance of declining and infrequent social participation as one of the risk factors for loneliness in old age [9, 10]. Contrary to this, sustained social and community participation, as well as frequent social contact with close others such as family members and social support, can protect against loneliness [8, 10]. There is, therefore, a need to identify sustainable, community-based solutions to foster social interactions and prevent loneliness in older adults.

In recent years, engagement with arts-based activities and venues has been increasingly used as a key ingredient of public health interventions [11, 12], leading to reports of multiple health and well-being benefits associated with cultural and arts engagement in older adulthood [13, 14]. Arts engagement within general population-based health research usually refers to broadly defined expressed or experienced human artistic creativity [14, 15] and is commonly conceptualised as participatory engagement with the arts (active creation of visual arts, drama, music or other art forms), and receptive arts engagement (attendance at arts-based events and venues such as museums, galleries, concert halls, and theatres). The majority of the evidence to date describes health and well-being benefits associated with participatory arts engagement for older adults [13, 14]. Nonetheless, frequent receptive arts engagement has been previously linked, among others, with lower odds of incident depressive symptoms and higher levels of happiness, life satisfaction, self-realisation and perceived independence in the English Longitudinal Study of Ageing (ELSA) dataset [16, 17] as well as lower odds of poor self-rated health and mortality in Swedish adult population-representative, prospective studies [18, 19].

The role of participatory and receptive arts engagement in preventing or alleviating loneliness among older adults, as well as facilitating bonding in new and existing relationships, has been also increasingly recognised [20, 21]. The most substantial body of evidence to date documents the role of music in preventing feelings of loneliness as well as sustaining social activity and independence in old age [22–26]. For instance, participation in community-based choir groups has been linked to a decrease in loneliness levels over a 2 year period [27], whilst making music such as singing in a choir or learning to make music has been also found to facilitate forming new social relationships and cultivating existing ones by providing opportunities for social affirmation, social support, and new forms of interaction [23, 25, 26, 28]. Studies involving adult populations further suggest that engagement with group-based musical and other creative activities increases the pace of social bonding and perceptions of closeness among participants, with some evidence suggesting a stronger effect for singing compared with other activities [29, 30], albeit inconsistently [31]. Others also demonstrated that visual art can be used to stimulate participation in a social network [32], and similar beneficial relational changes, such as increasing the number and closeness of social relationships, were observed for older adults participating in creative arts programmes [33] and painting workshops [34]. Most recently, museum-based programs for isolated older adults were also found to foster social inclusion by enabling social interactions and cultivation of meaningful relationships [21].

The evidence to date indicates that arts engagement in older age may serve as a multimodal intervention helping to prevent loneliness and enhance social interactions alongside other aspects of well-being and health. However, the majority of this evidence is based on small-scale and short-term intervention studies assessing the impact of participatory arts-based programmes run for a limited period of time and prone to significant self-selection bias. Receptive arts engagement should not be overlooked. In the UK there are over 2500 museums [35], 1300 theatres [36], and 4000 libraries [37] as well as 400 historic places [38], and 10,000 village halls [39] in England alone. These venues provide social spaces and opportunities to engage with the arts and leisure activities, and could be utilised as ‘assets’ to help reduce loneliness within communities. Yet, to the best of our knowledge, there are no previous large-scale, general population-based studies examining receptive arts engagement and its association with loneliness in older adulthood. To address this, we have used data from a large, nationally representative dataset of older adults aged over 50 years, the English Longitudinal Study of Ageing (ELSA), and investigated associations between frequency of visits to (i) the cinema, (ii) art galleries, exhibitions or museums, and (iii) the theatre, concerts, or the opera and odds of loneliness at baseline and 10 years later.

## Methods

### Data and study sample

We used data from the English Longitudinal Study of Ageing (ELSA), a large, ongoing longitudinal cohort study representative of the non-institutionalised English population of people aged 50 years and older on enrolment in 2002–2003 and designed as a stratified random sample of private households drawn originally from 1998, 1999, and 2001 Health Survey for England (HSE) [40]. For ELSA wave 1, participants born before 1 March 1952 and living in a private household in England participating in HSE, in which at least one person consented to follow-up, were considered eligible to participate [40]. Partners living in the same household as ‘core’ study member were also eligible to participate in the study. ELSA wave 1 household and individual response rates were 70% and 67%, respectively [40]. The participants were followed-up at 2-yearly intervals and the original sample was refreshed with additional HSE respondents to maintain the general population representativeness [40]. We specifically worked with data from ‘core’ ELSA members who took part in wave 2 (2004–2005) and wave 7 (2014–2015). Our analytical samples consisted of participants with complete arts engagement, covariate and loneliness data at wave 2 for cross-sectional analyses (*n* = 6222), and additionally complete loneliness data at wave 7 for longitudinal analyses (*n* = 3127).

### Loneliness

Loneliness was measured using the three-item short-form of the Revised UCLA Loneliness Scale which has been previously demonstrated to have good convergent and discriminant validity [41]. The three items assess how often the participants feel “left out”, “isolated from others”, and “lack companionship”. The answers are rated on a three-point scale—1 (hardy ever or never), 2 (some of the time), 3 (often)—and summed to produce a score ranging from 3 to 9, with higher scores indicating greater loneliness. Due to significant positive skew, the score was dichotomised to create a binary variable, grouping participants scoring 3–5 as “not lonely” and those scoring “6–9” as “lonely” in agreement with previous practice [42, 43].

### Arts engagement

Arts engagement was self-reported by the participants at wave 2 and consisted of three items asking about the frequency of visits to: (a) the cinema, (b) art galleries, exhibitions or museums, (c) the theatre, concerts, or the opera. Each arts engagement item was assessed on a five-point scale: 0 (never), 1 (less than once a year), 2 (once or twice a year), 3 (every few months), 4 (once a month or more). The two most frequent categories were combined for analyses due to a small sample size, creating an “every few months or more” category.

### Covariates

Variables considered likely to confound the associations between arts engagement and loneliness were measured at baseline (wave 2). Demographic covariates included age, gender, and ethnicity (coded as White and non-White as ELSA is > 98% white British). Socioeconomic status was assessed with highest educational attainment (categorised as: University degree or equivalent, including NVQ4–NVQ5; A level/higher education or equivalent including NVQ3; the General Certificate of Education incl. Ordinary level qualification (GCE/O level) or equivalent including NVQ2; other or no educational qualification), employment status (full-time, part-time, not in employment) and net non-pension wealth quintiles which measure the accumulation of assets over the lifespan and have been previously reported as the most salient socioeconomic position indicator in the ELSA cohort [44]. Health covariates included long-standing illness status (no long-standing illness, long-standing and non-limiting illness, long-standing and limiting illness), eyesight and hearing problems, as well as experiences of moderate or severe pain that could hinder one’s overall arts engagement. Additionally, participants registered as blind (*n* = 33) were excluded from the analyses due to possible different profiles of engagement in arts activities. Social factors included: coupled relationship status (in a couple vs. without a partner); a social contact variable derived as a composite score of frequency of contact (including contact over the phone, email, and face to face) with friends, children and wider relatives (coded as + 1 for each mode of contact and social tie, if contact occurred on a monthly basis or more frequently, with the score ranging 0–9); and a binary variable specifying engagement in any community activities (including being a member of a political party or environmental group, a tenants or neighbourhood watch group, a church or religious association, a charitable association, an education, arts or music class, a social club, a sports, gym or exercise class, or any other society).

### Statistical analysis

We investigated the association between three receptive arts engagement activities visits to (a) the cinema, (b) museums/galleries/exhibitions, and (c) theatre/concerts/opera—at baseline (wave 2) and odds of loneliness cross-sectionally (at wave 2) and longitudinally, a decade later (at wave 7). We used univariable (Model 1) and multivariable logistical regression models, adjusted for demographic and socioeconomic covariates (gender, age, ethnicity, highest educational attainment, employment status, and net non-pension wealth, Model 2), health variables (long-standing illness status, eyesight and hearing problems and pain, Model 3) and social factors (coupled relationships status, social contact score and engagement with community activities, Model 4). Model 5 additionally adjusted for wave 2 loneliness in the longitudinal analysis to establish if the associations between the odds of loneliness and arts engagement remain after controlling for baseline loneliness.

All analyses were weighted using baseline cross-sectional weights to minimise bias from differential non-response amongst key subgroups. Participants with missing information on arts engagement, covariates and loneliness at wave 2 were excluded from cross-sectional analyses, yielding a final cross-sectional analytical sample of 6222. Out of these, 3095 participants who missed ELSA wave 7 and did not provide wave 7 loneliness data were excluded from the longitudinal analytical sample, yielding a final longitudinal analytical sample of *n* = 3127. As a sensitivity analysis, missing covariate data were imputed using multiple imputation by chained equations; however, the results of the analyses did not vary materially between complete case and imputed datasets, hence the findings presented here are based on the complete case dataset.

## Results

The analytical samples used for cross-sectional and longitudinal analyses are described in Table 1. The sample included in the longitudinal sample was predominantly White British, the mean age was 62.5 years of age and 44.9% of participants were men. Approximately 50% of participants reported no formal education or GCE/O level as the highest educational qualification and 54.8% were not in employment. Just over 50% of participants reported having long-standing illness, 8.0% had eyesight problems, 16.5% had hearing problems and 5.4% reported pain. Approximately 75% were in a coupled relationship and 79.1% were engaged in community and civic activities. On average participants scored 4.8 out of 9 on the social contact scale. Most frequent arts engagement, taking place every few months or more often, was reported by 25.9%, 19.8% and 27.3% of participants for visits to the cinema, galleries/exhibitions/museums, and theatre/concerts/opera, respectively. No arts engagement was reported by 30.0%, 29.0% and 24.5% of participants for visits to the cinema, galleries/exhibitions/museums, and theatre/concerts/opera, respectively. Of 6222 participants at wave 2, 18.9% (*n* = 1178) were lonely, of those 47.8% remained lonely and 52.2% no longer reported loneliness at wave 7. Just over 16% (*n* = 510) of participants reported loneliness at wave 7.

**Table 1:** Descriptive characteristics of participants included in the complete cases analyses of cross-sectional sample (*n* = 6222) and longitudinal sample (*n* = 3127), and those excluded from longitudinal analysis (*n* = 3095)

Covariates at wave 2	Cross-sectional sample (*n* = 6222)	Longitudinal sample (*n* = 3127)	Excluded sample (*n* = 3095)	*p*
Gender: male, *n* (%)	2896 (46.5%)	1405 (44.9%)	1491 (48.2%)	0.010
Age, mean (SD)	65.60 (9.46)	62.50 (7.15)	68.72 (10.43)	< 0.001
Ethnicity: Non-white, *n* (%)	87 (1.4%)	36 (1.2%)	51 (1.7%)	0.10
Education, *n* (%)				
Degree	882 (14.2%)	552 (17.6%)	330 (10.7%)	< 0.001
A level/higher education	1858 (29.9%)	1018 (32.6%)	840 (27.1%)	
GCE and O level	1161 (18.7%)	685 (21.9%)	476 (15.4%)	
No qualification	2321 (37.3%)	872 (27.9%)	1449 (46.8%)	
Employment status, *n* (%)				
Not in employment	4009 (64.4%)	1712 (54.8%)	2297 (74.2%)	< 0.001
Full time ≥ 35 h/week	1275 (20.5%)	804 (25.7%)	471 (15.2%)	
Part time	938 (15.1%)	611 (19.5%)	327 (10.6%)	
Long-standing illness, *n* (%)				
No long-standing illness	2755 (44.3%)	1532 (49.0%)	1223 (39.5%)	< 0.001
Long-standing, not limiting illness	1403 (22.5%)	770 (24.6%)	633 (20.5%)	
Long-standing and limiting illness	2064 (33.2%)	825 (26.4%)	1239 (40.0%)	
Eyesight problems: Yes, *n* (%)	720 (11.6%)	249 (8.0%)	471 (15.2%)	< 0.001
Hearing problems: Yes, *n* (%)	1260 (20.3%)	515 (16.5%)	745 (24.1%)	< 0.001
Pain: Yes, *n* (%)	430 (6.9%)	169 (5.4%)	261 (8.4%)	< 0.001
Coupled relationship status: Yes, *n* (%)	4418 (71.0%)	2357 (75.4%)	2061 (66.6%)	< 0.001
Social contact score (0–9), mean (SD)	4.63 (1.85)	5.82 (1.81)	4.44 (1.87)	< 0.001
Community engagement: Yes, *n* (%)	4622 (74.3%)	2473 (79.1%)	2149 (69.4%)	< 0.001
Frequency of arts engagement				
Cinema, *n* (%)				
Never	2520 (40.5%)	937 (30.0%)	1583 (51.2%)	< 0.001
Less than once a year	1418 (22.8%)	776 (24.8%)	642 (20.7%)	
Once or twice a year	1018 (16.4%)	603 (19.3%)	415 (13.4%)	
Every few months or more	1266 (20.3%)	811 (25.9%)	455 (14.7%)	
Galleries/exhibitions/museums, *n* (%)				
Never	2451 (39.4%)	908 (29.0%)	1534 (49.9%)	< 0.001
Less than once a year	1562 (25.1%)	883 (28.3%)	679 (21.9%)	
Once or twice a year	1220 (19.6%)	716 (22.9%)	504 (16.3%)	
Every few months or more	989 (15.9%)	620 (19.8%)	369 (11.9%)	
Theatre/concerts/opera, *n* (%)				
Never	2133 (34.3%)	766 (24.5%)	1367 (44.2%)	< 0.001
Less than once a year	1305 (21.0%)	699 (22.4%)	606 (19.6%)	
Once or twice a year	1374 (22.1%)	807 (25.8%)	567 (18.3%)	
Every few months or more	1410 (22.6%)	855 (27.3%)	555 (17.9%)	
Loneliness (score ≥ 6) at wave 2	1178 (18.9%)	–	–	
Loneliness (score ≥ 6) at wave 7	–	510 (16.3%)	668 (21.6%)	< 0.001

*P* values correspond to statistical differences between samples included and excluded from the longitudinal analysis. Data comes from ELSA study waves 2 (2004–2005) and 7 (2014–2005)

### Cross-sectional analysis

The results from cross-sectional logistic regression models are presented in Table 2. We found dose response, negative associations between odds of loneliness and engagement with cinema, galleries/exhibitions/museums and theatre/concerts/opera. In the final, fully-adjusted models, we found that engaging with cinema every few months or more often, compared with never, was associated with 26% lower odds of loneliness (OR = 0.74, 95% confidence interval (CI) 0.59–0.93, *p* = 0.009). Participants reporting visits to galleries/exhibitions/museums every few months or more often and once or twice a year had, respectively, 26% (OR = 0.74, 95% CI 0.57–0.94, *p* = 0.016) and 22% (OR = 0.78, 95% CI 0.62–0.97, *p* = 0.024) lower odds of loneliness compared with those who reported no engagement. Participants reporting visits theatre/concerts/opera every few months or more often and once or twice a year had, respectively, 33% (OR = 0.67, 95% CI 0.53–0.84, *p* = 0.001) and 23% (OR = 0.77, 95% CI 0.62–0.95, *p* = 0.013) lower odds of loneliness compared with those who reported no engagement.

**Table 2:** Results from logistic regression models examining the association between baseline (2004–2005) arts engagement and odds of loneliness (UCLA-3 item ≥ 6) at ELSA wave 2 (2004–2005), *n* = 6222

	Model 1			Model 2			Model 3			Model 4		
	OR	95% CI	*p*	OR	95% CI	*p*	OR	95% CI	*p*	OR	95% CI	*p*
Frequency of visits to the cinema (reference: never)												
≤ Once a year	0.64	0.54–0.76	< 0.001	0.84	0.71–1.01	0.068	0.91	0.76–1.10	0.32	0.98	0.81–1.18	0.81
Once or twice a year	0.56	0.46–0.68	< 0.001	0.78	0.63–0.97	0.023	0.88	0.71–1.09	0.25	0.91	0.73–1.14	0.44
≥ Every few months	0.44	0.37–0.54	< 0.001	0.64	0.52–0.80	< 0.001	0.73	0.59–0.91	0.005	0.74	0.59–0.93	0.009
Frequency of visits to galleries/exhibitions/museums (reference: never)												
≤ Once a year	0.53	0.45–0.62	< 0.001	0.69	0.58–0.83	< 0.001	0.78	0.65–0.94	0.007	0.83	0.68–1.01	0.63
Once or twice a year	0.46	0.38–0.56	< 0.001	0.65	0.53–0.79	< 0.001	0.74	0.60–0.91	0.005	0.78	0.62–0.97	0.024
≥ Every few months	0.44	0.36–0.55	< 0.001	0.65	0.51–0.82	< 0.001	0.75	0.59–0.95	0.016	0.74	0.57–0.94	0.016
Model 1: Frequency of visits to the theatre/concerts/opera (reference: never)												
≤ Once a year	0.60	0.50–0.72	< 0.001	0.76	0.63–0.92	0.004	0.84	0.69–1.02	0.08	0.88	0.72–1.07	0.21
Once or twice a year	0.48	0.40–0.57	< 0.001	0.63	0.52–0.77	< 0.001	0.72	0.59–0.88	0.001	0.77	0.62–0.95	0.013
≥ Every few months	0.39	0.33–0.48	< 0.001	0.55	0.45–0.69	< 0.001	0.65	0.52–0.81	< 0.001	0.67	0.53–0.84	0.001

*Model 1* univariate, *Model 2* demographic factors: Model 1 + gender, age, ethnicity, highest educational attainment, employment status, and net non-pension wealth, *Model 3* health factors: Model 2 + health variables: eyesight and hearing problems, experiences of pain and long-standing illness status, *Model 4* social factors: Model 3 + social contact, romantic relationship status, and engagement with community activities

### Longitudinal analysis

The results from longitudinal logistic regression models are presented in Table 3. We found no association between frequency of engagement with cinema and odds of loneliness after adjusting for covariates. We found a dose response, negative association between engagement with galleries/exhibitions/museums and odds of loneliness, which remained after all adjustments. In the final, fully-adjusted model, engaging with galleries/exhibitions/museums every few months or more often, compared with never, was associated with 32% lower odds of reporting loneliness at wave 7 (OR = 0.68, 95% CI 0.48–0.95, *p* = 0.025) and engaging once or twice a year was associated with 26% lower odds of reporting loneliness at wave 7 (OR = 0.74, 95% CI 0.54–1.01, *p* = 0.055). We also found evidence, albeit less consistent, to suggest that more frequent engagement with the theatre/concerts/opera was associated with lower odds of loneliness over time. In the fully-adjusted model, engaging with the theatre/concerts/opera once or twice a year, compared with never, was associated with 31% lower odds of reporting loneliness at wave 7 (OR = 0.69, 95% CI 0.50–0.95, *p* = 0.021). Further, compared with no engagement with the theatre/concerts/opera, engagement every few months or more often ceased to be significantly associated with odds of loneliness once social factors were accounted for, whilst engagement on a less than once a year basis remained associated with lower odds of loneliness (Model 4, OR = 0.75, 98% CI 0.56–0.99, *p* = 0.046).

**Table 3:** Results from logistic regression models examining the association between baseline (ELSA wave 2, 2004–2005) arts engagement and odds of loneliness (UCLA-3 item ≥ 6) 10 years later (ELSA wave 7, 2014–2015), *n* = 3127

	Model 1			Model 2			Model 3			Model 4			Model 5		
	OR	95% CI	*p*	OR	95% CI	*p*	OR	95% CI	*p*	OR	95% CI	*p*	OR	95% CI	*p*
Frequency of visits to the cinema (reference: never)															
≤ Once a year	0.83	0.65–1.06	0.13	1.03	0.80–1.33	0.82	1.07	0.88–1.49	0.33	1.14	0.88–1.49	0.33	1.15	0.87–1.54	0.31
Once or twice a year	0.61	0.46–0.81	0.001	0.79	0.59–1.07	0.13	0.85	0.66–1.22	0.49	0.90	0.66–1.22	0.49	0.91	0.66–1.27	0.59
≥ Every few months	0.60	0.46–0.77	< 0.001	0.84	0.64–1.10	0.20	0.91	0.72–1.27	076	0.96	0.72–1.27	0.76	0.96	0.78–1.41	0.73
Frequency of visits to galleries/exhibitions/museums (reference: never)															
≤ Once a year	0.61	0.48–0.77	< 0.001	0.71	0.56–0.91	0.007	0.77	0.60–0.99	0.038	0.82	0.63–1.06	0.13	0.86	0.65–1.13	0.23
Once or twice a year	0.50	0.39–0.66	< 0.001	0.63	0.48–0.83	0.001	0.68	0.51–0.90	0.007	0.71	0.53–0.94	0.020	0.74	0.54–1.01	0.055
≥ Every few months	0.41	0.31–0.55	< 0.001	0.57	0.42–0.79	0.001	0.61	0.44–0.84	0.003	0.64	0.46–0.89	0.008	0.68	0.48–0.95	0.025
Model 1: Frequency of visits to the theatre/concerts/opera (reference: never)															
≤ Once a year	0.58	0.44–0.75	< 0.001	0.66	0.50–0.88	0.004	0.71	0.54–0.94	0.018	0.75	0.56–0.99	0.046	0.75	0.55–1.02	0.07
Once or twice a year	0.48	0.37–0.63	< 0.001	0.58	0.44–0.77	< 0.001	0.63	0.47–0.84	0.002	0.68	0.51–0.92	0.012	0.69	0.50–0.95	0.021
≥ Every few months	0.49	0.38–0.63	< 0.001	0.64	0.48–0.84	0.001	0.70	0.53–0.93	0.015	0.76	0.57–1.03	0.08	0.84	0.61–1.15	0.27

*Model 1* univariate, *Model 2* demographic factors: Model 1 + gender, age, ethnicity, highest educational attainment, employment status, and net non-pension wealth, *Model 3* health factors: Model 2 + health variables: eyesight and hearing problems, experiences of pain and long-standing illness status, *Model 4* social factors: Model 3 + social contact, romantic relationship status, and engagement with community activities, *Model 5* Model 4 + baseline loneliness score

Finally, it is worth to note that the longitudinal analytical sample was skewed towards participants who were female, younger, employed, more educated, in good health, coupled relationships, reporting greater social, community and arts engagement as well as less likely to be lonely at wave 2 (Table 1).

## Discussion

This study using a large sample of the ELSA dataset found that frequent receptive arts engagement was associated with lower odds of loneliness contemporaneously, and a decade later. In cross-sectional fully-adjusted analyses at ELSA wave 2, we found that frequent engagement with all three activities (cinema, galleries/exhibitions/museums, theatre/concerts/opera) was associated with lower odds of loneliness. Longitudinally, we found that frequent visits to galleries, exhibitions and museums in particular, as well as to some degree frequent visits to the theatre, concerts and opera may be modestly protective against loneliness over time. We found that these associations were independent of baseline loneliness score and a number of sociodemographic, socioeconomic and health factors, plus other forms of social engagement such as frequency of contact with friends, family, children and participation in different community activities.

Our longitudinal findings are in line with previous research focusing on museum and gallery attendance. Todd et al. reported on a series of museum programmes designed to engage socially isolated older adults in participatory activities including object handing, participatory arts and singing. These programmes were found to aid perceived social inclusion by providing a context for social interactions and a safe and stimulating space that enabled positive change for the individual such as enhanced self-esteem and positive interpersonal experiences including opportunities for social interactions and cultivating relationships [21]. Art gallery-based interventions for people with dementia and their careers have been also found to foster feelings of social inclusion as well as emotional closeness in pairs [45]. Similarly, the monthly Meet Me at MoMA programme, run at the Museum of Modern Art in New York for people in the early and middle stage of Alzheimer’s disease and their carers was observed to support and facilitate shared experiences as well as being an inherently social experience [46]. Indeed, Camic and Chatterjee argue that museums and art galleries play an important social role in the health and well-being of communities [11]. Other intervention studies also demonstrate that participatory engagement with visual arts within community setting helped older adults to overcome prolonged social isolation and facilitated socialising with others [32, 34].

We found lesser evidence of a longitudinal protective association for older adults’ engagement with the theatre, concerts and opera and no longitudinal association between engagement with the cinema and loneliness. While all arts activities measured in this study could be described as receptive forms of arts engagement, i.e. attending rather than participating at arts events and venues, the more passive and least interactive activities—including cinema—may be the least conducive to counteracting loneliness in older adults. Indeed, it has been suggested that participatory arts, involving active involvement in arts-based activities, may be most effective at tackling loneliness and assisting isolated older adults in regaining their confidence to reconnect with others [20]. However, no empirical comparisons of participatory and receptive art engagement and loneliness have been produced to date. Art-based participatory interventions are usually run for a limited time and require a dedicated and skilled programme leader, which poses numerous sustainability challenges. Here, we demonstrate that frequent self-directed arts attendance may also help to protect older adults from loneliness. Nonetheless, specific programmes and skilled staff members are necessary to facilitate greater access and social inclusion required to foster positive social outcomes, particularly for isolated and vulnerable older adults such as people with dementia and their carers [21, 45]. It is also acknowledged that the measure of arts engagement used in ELSA was not detailed enough to preclude the possibility of more participatory forms of engagement during participants’ arts activities. For instance, the study participants may have visited cultural venues such as museums and galleries to take part in participatory workshops or activities such as educational classes. Further research is needed to investigate the differences between art forms and cultural venues in opportunities for social inclusion and shared experiences facilitating positive social contact of older adults.

Multiple beneficial social outcomes associated with arts engagement have been identified that can help to elucidate its role in counteracting loneliness [47]. For instance, engagement with music has been reported by older adults as a direct coping strategy for avoiding and alleviating feelings of loneliness [22, 48]. In fact, regulation of feelings of loneliness is one of the motivations for listening to music frequently reported in the wider literature [49, 50]. Arts engagement is often a social activity in itself as people tend to attend arts-based events and venues accompanied and for the shared positive social experiences [21, 51]. The atmosphere of social inclusion present in the art spaces can be particularly conducive to older adults who may need additional support to re-connect with others, particularly in overcoming the challenge of prolonged isolation and/or low self-esteem and confidence [21, 33]. Arts-based activities have been further found to facilitate the creation of new social relationships [21, 26, 32, 33] and increase the pace of bonding and closeness in relationships [30, 33, 52]. Relationships created or strengthened as part of engaging with the arts, such as making music in local community groups can increase access to different forms of support, such as peer or informational support, and act as a source of social affirmation [23, 26]. Arts-based activities have been also shown to increase closeness of existing family and peer relationships by extending the usual types of shared activities and interactions [25, 34, 51] as well as to boost feelings of belonging on a larger scale by increasing connectedness in the wider community [26, 53]. Consequently, arts-based engagement has the potential to facilitate several of the intervention strategies identified previously [54] as relevant to reducing loneliness: improving social skills, enhancing social support, increasing opportunities for social contact, and to some, yet limited, extent addressing maladaptive social cognition [21]. Further research exploring how receptive arts activities specifically may protect older adults against loneliness, and how they compare to participatory arts activities as well as other leisure and hobby activities such as sports, is still warranted. As arts engagement is often a shared experience, future research is needed to understand the interplay between the social and creative processes occurring in encounters with the arts. More research is also needed to understand barriers and facilitators of arts engagement in lonely and isolated adults.

## Strengths and limitations

The study is the first to address the association between receptive arts engagement and loneliness in old age using a large, general population-based cohort study with rich information on health and social covariates, and a longitudinal design. Nonetheless, it also includes several limitations. Our estimates are likely biased by non-random attrition commonly found in observational, longitudinal research. We found that participants included in the final analytical sample were younger, more educated, healthier and more socially engaged. These patterns of attrition might have increased the study’s vulnerability to residual confounding by socioeconomic and health status differences in access and engagement with arts activities. We further found that participants excluded from the analytical sample were more likely to report no arts engagement and loneliness at baseline, which could further bias the findings, yet likely leading to an underestimation of the association.

This study also suffers from bias resulting from measurement issues related to arts engagement, which requires longer and more comprehensive scales in order to shed light on the plethora of ways in which older adults can engage in arts activities. Compared with lists of activities measured in other surveys, such as Understanding Society, the number of arts-based activities included in ELSA is limited and restricted to engagement in receptive and usually ticketed arts activities occurring in government-supported or commercial cultural venues, thus likely leading to underestimated and biased representation of arts engagement in the population [55]. The conceptualisation of arts engagement in ELSA is not, however, atypical in its the omission of more popular forms of cultural participation, including participatory arts and thus bias towards “highbrow interpretation” of what constitutes arts engagement [55–57].

## General conclusions

Our study has shown that frequent arts engagement in older adults is associated with reduced odds of loneliness contemporaneously, and 10 years later. We find that frequent visits to galleries, exhibitions and museums in particular appear to be protective against loneliness in older adults over time, independently of baseline loneliness level and various demographic, socioeconomic, health and social factors. If replicated in future studies, these findings would suggest that those who manage places and spaces for arts engagement should, at the least, be aware of the potential for their venues to facilitate shared experiences and positive social interactions and, even better, direct their resources and programming toward facilitating such opportunities. Future research is needed to understand the mechanisms through which different arts activities, in particular receptive arts engagement, can contribute to preventing and alleviating feelings of loneliness and facilitating social connectedness among older adults.
